# Demand and capacity to integrate pelvic organ prolapse and genital fistula services in low-resource settings

**DOI:** 10.1007/s00192-018-3561-2

**Published:** 2018-02-06

**Authors:** Vandana Tripathi, Sohier Elneil, Lauri Romanzi

**Affiliations:** 1 Fistula Care Plus Project at EngenderHealth, 440 9th Avenue, 12th Floor, New York, NY 10001 USA; 2 University College London Hospital and National Hospital for Neurology and Neurosurgery, 235 Euston Road, London, NW1 2PG UK

**Keywords:** Genital fistula, Global surgery, Health systems research, Integration, Low- and middle-income countries, Pelvic organ prolapse

## Abstract

**Introduction and hypothesis:**

There is a need for expanded access to safe surgical care in low- and middle-income countries (LMICs) as illustrated by the report of the 2015 Lancet Commission on Global Surgery. Packages of closely-related surgical procedures may create platforms of capacity that maximize impact in LMIC. Pelvic organ prolapse (POP) and genital fistula care provide an example. Although POP affects many more women in LMICs than fistula, donor support for fistula treatment in LMICs has been underway for decades, whereas treatment for POP is usually limited to hysterectomy-based surgical treatment, occurring with little to no donor support. This capacity-building discrepancy has resulted in POP care that is often non-adherent to international standards and in non-integration of POP and fistula services, despite clear areas of similarity and overlap. The objective of this study was to assess the feasibility and potential value of integrating POP services at fistula centers.

**Methods:**

Fistula repair sites supported by the Fistula Care *Plus* project were surveyed on current demand for and capacity to provide POP, in addition to perceptions about integrating POP and fistula repair services.

**Results:**

Respondents from 26 hospitals in sub-Saharan Africa and South Asia completed the survey. Most fistula centers (92%) reported demand for POP services, but many cannot meet this demand. Responses indicated a wide variation in assessment and grading practices for POP; approaches to lower urinary tract symptom evaluation; and surgical skills with regard to compartment-based POP, and urinary and rectal incontinence. Fistula surgeons identified integration synergies but also potential conflicts.

**Conclusions:**

Integration of genital fistula and POP services may enhance the quality of POP care while increasing the sustainability of fistula care.

**Electronic supplementary material:**

The online version of this article (10.1007/s00192-018-3561-2) contains supplementary material, which is available to authorized users

## Introduction

Global health programming in low- and middle-income countries (LMICs) has historically often involved silo-funded efforts, both for nonsurgical and surgical conditions. Recent trends in non-surgical health programs, however, reflect a new vision of integrated care that optimizes the economies of both the financial scale and clinical scope of services provided [1, 2]. The past three decades have yielded substantive progress in a number of non-surgical global health priorities in LMICs, such as under-five mortality. Comparable progress has been lacking in the field of surgery and anesthesia, but recent developments suggest potential for change. On 26 May 2015, the World Health Assembly released Resolution 68.15, entitled: “Strengthening emergency and essential surgical care and anaesthesia as a component of universal health coverage” [3]. This resolution recognizes the impact on health and disability of surgically treatable conditions and describes improved local surgical capacity as a “highly cost-efficient solution to the global burden of disease” [3]. Also in 2015, the report of the Lancet Commission on Global Surgery estimated that five billion people lack access to safe, affordable surgical care, not only because of gaps in clinical skills, but also deficits in physical infrastructure, personnel, and equipment [4]. To achieve the scale and scope required in capacity building support for global surgery, the Lancet Commission advanced the concept of “Bellwether Procedures”—laparotomy, treatment of open fractures, and cesarean section—whose consistent provision is an indicator of adequate surgical service provision to a community, in addition to indicating a facility primed for expansion of surgical service delivery [4].

As noted above, the few global initiatives for surgical services, outside of the strengthening of cesarean delivery integrated into maternal health systems, have generally been “siloed,” i.e., restricted to services for single clinical conditions, with resources mobilized only to provide a single type of operation in remote, resource-constrained communities. Single-issue surgical service initiatives in LMICs have targeted conditions such as clubfoot, cleft palate, cataracts, hydrocephalus, and genital fistulas [5–8].

Now, integrating expansion of surgical service delivery can mirror trends in broader global health programming to move away from silo-funded activities. In the emerging global surgery thinking, packages of closely-related surgical procedures may provide platforms of capacity that maximize impact in low-resource settings. In this vein, facilities providing access for fistula repair surgery may be the most efficient environments for expansion of services to include surgical care for pelvic organ prolapse (POP) and incontinence not caused by genital fistula, a paradigm first described in Walker and Gunasekera’s 2011 call to action regarding POP and incontinence [9].

Genital fistula has received significant attention in the past decade, with donors and affected countries expanding access to surgical repair. Although POP occurs worldwide, women in LMICs suffer from untreated POP for longer, with quality of life consequences rivaling those of fistulas [10, 11]. Although POP is estimated to affect 20% of parous women in LMICs, population-level data remain sparse [9]. POP treatment in low-resource settings is limited and often non-adherent to international standards for terminology, evaluation, and management of pelvic floor disorders [11, 12]. The paucity of data in LMICs makes it difficult to make precise estimates of service needs. However, the high burden of POP and inadequate level of treatment is also corroborated by the experiences of surgeons running “fistula camps” in under-resourced settings, who report that a significant proportion of women who attend do not have fistulas but rather POP and other forms of incontinence [13, 14].

Many women with POP require surgical treatment; these procedures require the same instruments and dissection skills as those for fistula patients. In response to the unmet need for POP care in the fistula community and the capacity for expanded services within the academic sector, master fistula surgeons in Ethiopia, Ghana, and Nigeria have launched accredited female pelvic medicine or urogynecology training programs that integrate training and service provision for fistula, POP, and non-fistula incontinence [15, 16]. The similarity of clinical skills needed for both POP and fistula treatment favor such joint approaches to training, funding, capacity building, and service delivery.

The USAID-funded Fistula Care *Plus* (FC+) Project at EngenderHealth partners with over 30 surgical fistula repair sites in Bangladesh, the Democratic Republic of Congo (DRC), Niger, Nigeria, Togo, and Uganda. The objective of this study was to assess the feasibility and potential value of integrating POP services at fistula centers, an example of bundling closely related surgical services. We hypothesized that there is both demand and capacity for such integration.

## Materials and methods

The FC+ Project conducted a survey of POP need and capacity at project-supported fistula treatment sites in August–September 2015. Survey questions addressed current demand for POP services, surgical and nonsurgical treatment capacity for POP, methods of POP and lower urinary tract symptom (LUTS) evaluation, POP and incontinence surgical skill sets, and anticipated synergies and conflicts related to potential integration of POP care into fistula centers. The survey consisted of 13 modules, described in Table 1. The survey required 30–45 min to complete. The survey was circulated through Survey Monkey for completion by one respondent per fistula treatment site. The full survey instrument is provided as a Supplementary Material, with a preview version also available online [17]. As the survey elicited only key informant data from health professionals, it was classified as nonhuman subjects’ research, and thus did not require Institutional Review Board approval.

**Table 1:** Clinician survey modules and questions

Module topic	Number of questions
Facility and provider overview	8
Current fistula services	7
Fistula backlog	11
POP service demand	8
POP evaluation	7
Nonsurgical POP management	5
Surgical POP management	
Cystoscopy	2
Vaginal surgery	3
Abdominal surgery	2
Surgery for concomitant urinary incontinence	3
Surgery for concomitant rectal incontinence	1
Capacity to expand POP evaluation and management	4
POP integration synergies and conflicts	11

## Results

Survey responses were received from 26 fistula treatment sites in Bangladesh (8), DRC (5), Niger (3), Nigeria (8), and Uganda (2). Facility respondents were primarily obstetrician-gynecologists and general surgeons.

**Fig. 1:**
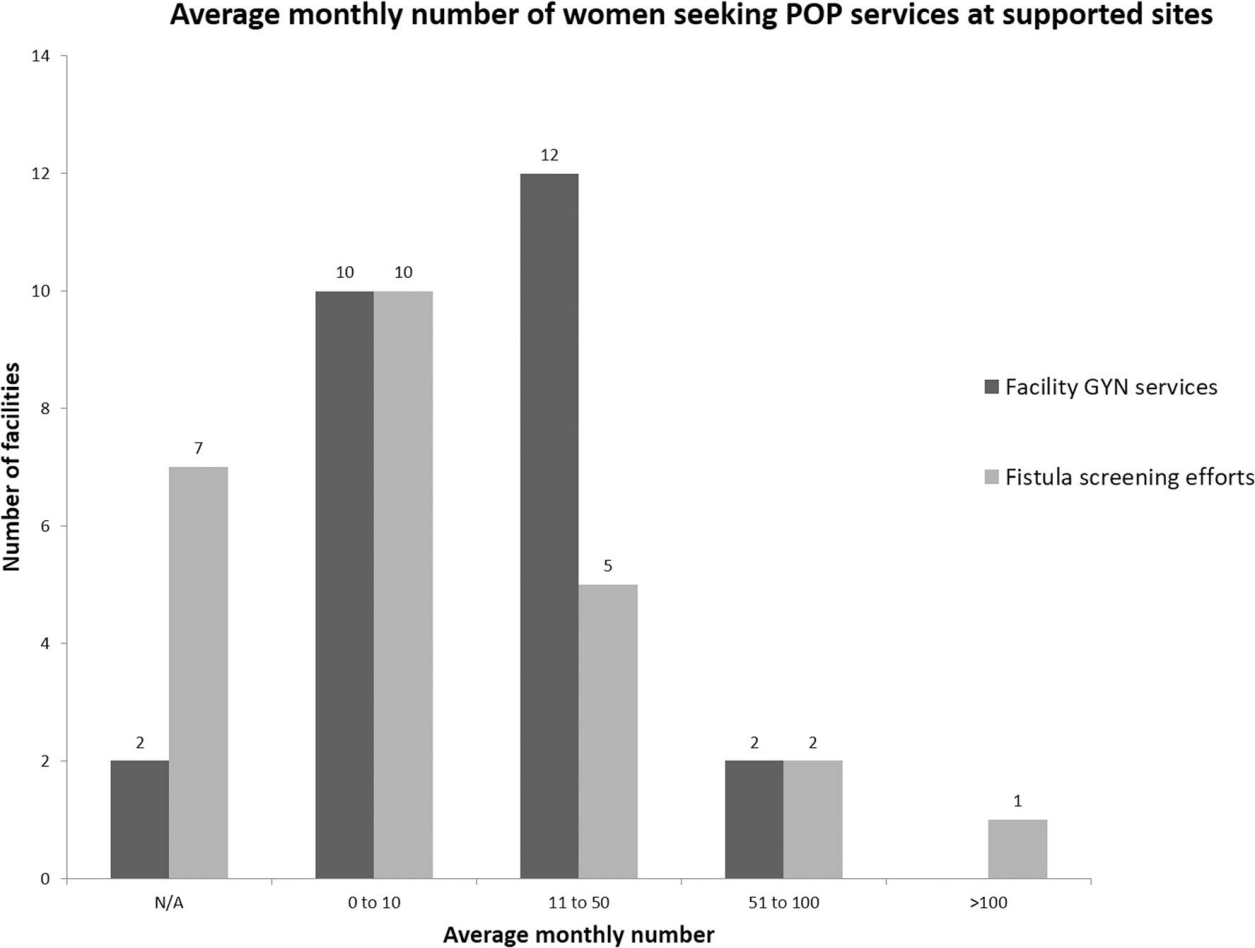
Average monthly number of women seeking POP services at fistula centers

Twenty-four (92%) sites report a demand for POP services. Most report that women arrive seeking POP care each month at both fistula screening efforts and facility-based gynecological services (Fig. 1). Although 22 facilities (85%) provide POP treatment, most do not fully meet the need. Fifteen facilities (58%) report that patient need for POP services is not met or only partially met. Yet, most facilities (73%) report that they do not refer externally for POP services. Among sites that do refer, respondents describe needing to send POP patients to facilities up to 320 km away.

Twenty-one facilities reported on their assessment processes for POP and incontinence. Of these, 15 (71%) adhere to the preferred practice of compartment-based assessment of prolapse anatomy, i.e., determining whether the prolapse is located in the uterus or top of the vagina (apex compartment), front wall of the vagina (anterior compartment), or back wall (posterior compartment) [18]. Seven centers (33% of those reporting) do not grade POP cases, whereas 11 centers either use POP-Q (29%) or Baden–Walker (24%), the main internationally accepted systems for staging degree of prolapse [19]. One center uses both POP-Q and Baden–Walker, and 2 rely on the degree of prolapse to grade POP cases.

Assessment of LUTS primarily relies on history-taking. Only 3 facilities (14%) reporting on LUTS assessment procedures routinely use bladder diary and 2 (10%) routinely use the pad test. Under half (43%) of facilities have both the skills and materials for simple cystometrics and no facility has both the skills and materials for multichannel cystometrics. However, most facilities (75%) use full bladder/reduction of POP/cough test to assess for stress urinary incontinence among POP patients. Eight (36%) facilities have access to cystoscopy, which is primarily used for diagnosis and ureteric catheterization.

Of facilities reporting on nonsurgical POP management, 11 (50%) provide physical therapy instructions, but only 6 (27%) report that all or most patients return for evaluation. Seven of the reporting facilities (32%) provide pessary management of POP. However, most report providing 5 or fewer patients with pessaries per month and only 3 report reliable pessary procurement.

The majority of facilities currently conduct most common apex, anterior, and posterior vaginal surgical procedures (Table 2). Fewer than half provide most abdominal surgical procedures (Table 3) or procedures to manage concomitant urinary incontinence (Table 4).

**Table 2:** Current practice of vaginal surgical POP procedures at fistula centers (*n* = 19)

Procedure	Facility conducts	Facility does not conduct
Apex		
Uterosacral cuff or vault suspension^a^	7	12
Uterosacral hysteropexy^a^	8	11
Sacrospinous vault or uterine suspension^b^	10	9
Enterocele repair^b^	14	5
Anterior		
Colporrhaphy cystocele repair^b^	18	1
Vaginal paravaginal cystocele repair^b^	13	6
Posterior		
Levatorplasty rectocele repair^a^	9	11
Site-specific rectocele repair^b^	14	6
Perineorrhaphy^b^	19	1
Perineoplasty^b^	12	8

^a^Procedures currently performed at fewer than half of responding facilities

^b^Procedures currently performed at more than half of responding facilities

**Table 3:** Current practice of abdominal surgical POP procedures at fistula centers (*n* = 20)

Procedure	Facility conducts	Facility does not conduct
Apex		
Uterosacral vault suspension^a^	9	11
Uterosacral hysteropexy^a^	6	14
Sacro-colpopexy^a^	8	12
Sacro-hysteropexy^a^	5	15
Anterior		
Paravaginal repair cystocele^a^	7	13

^a^Procedures currently performed at fewer than half of responding facilities

^b^Procedures currently performed at more than half of responding facilities

**Table 4:** Current practice of surgical procedures for concomitant incontinence (urinary and rectal) at fistula centers (*n* = 20)

Procedure	Facility conducts	Facility does not conduct
Vaginal		
Urethropexy (Kelly plication) ^b^	11	9
Anal sphincteroplasty^b^	14	6
Abdominal		
Urethropexy (Burch procedure) ^a^	4	16
Combined		
Rectus fascia autologous sling^a^	3	17
Fascia lata autologous sling^a^	1	18
Other for urinary incontinence^a^	2	18

^a^Procedures currently performed at fewer than half of responding facilities

^b^Procedures currently performed at more than half of responding facilities

**Fig. 2:**
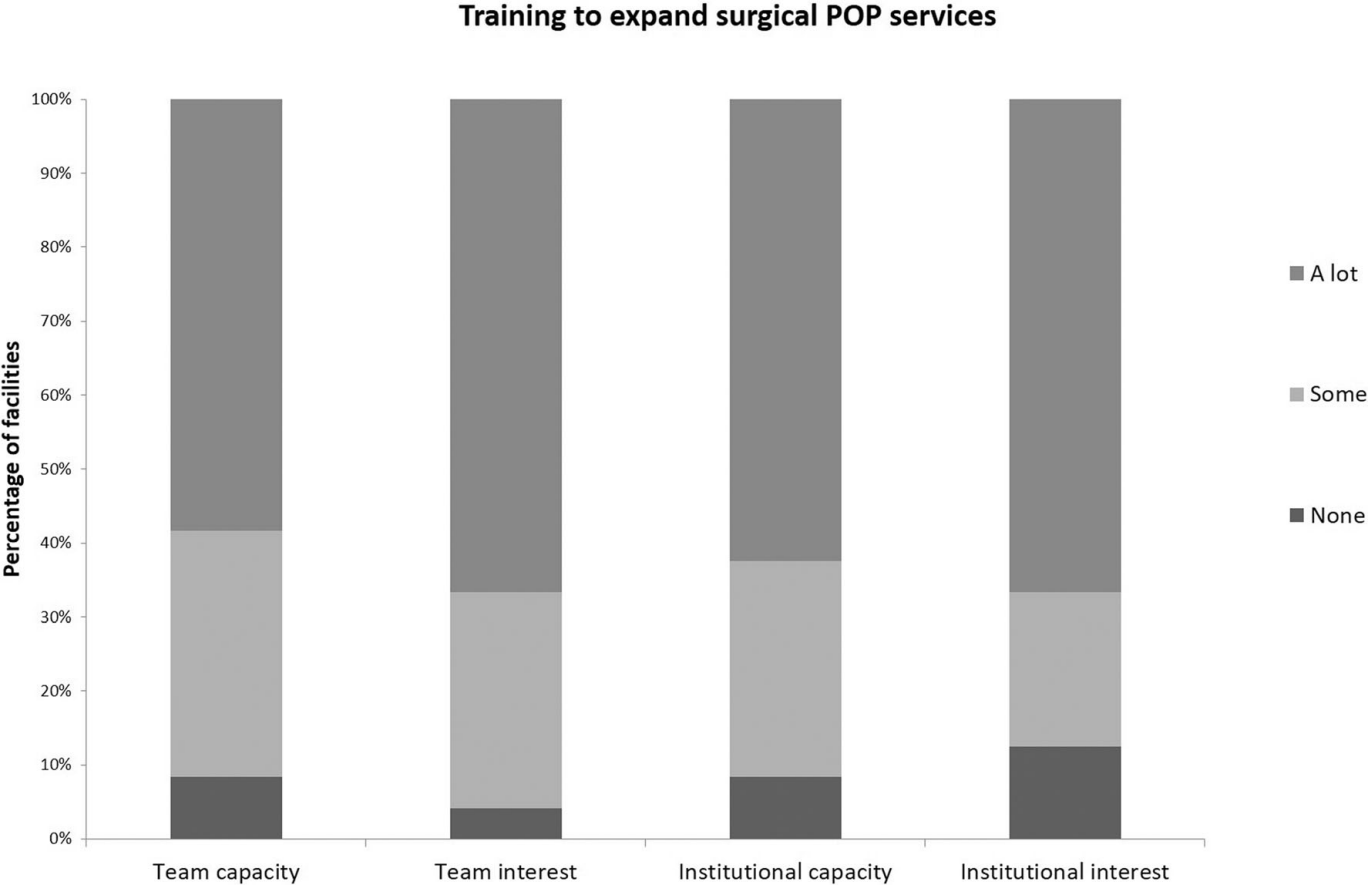
Interest and capacity at fistula centers to expand surgical POP services through training

Fistula surgeons report a high level of interest expanding and improving POP services, both surgical and nonsurgical (Fig. 2). However, several responding centers expressed concerns about the capacity of the facility to absorb such services. Respondents were asked to identify synergies and conflicts related to integrated POP and fistula services across the following topics: human resources, access/availability of camps/routine fistula services, development of surgical skills, infrastructure, equipment, expendable supplies/medication, data management systems, quality assurance/improvement (QA/QI), prevention, community engagement, and referral mechanisms. Overall, clinicians identified twice as many potential synergies as potential conflicts (Table 5). The topic with the most synergies identified was development of surgical skills, following closely by QA/QI and infrastructure. Notably, infrastructure was also the topic with the most conflicts identified, followed closely by expendable supplies/medication. Examples of specific potential synergies between fistula and POP care identified include: similar staff, infrastructure, equipment, and supplies needed; potential for teambuilding and to improve provider morale; overlapping surgical skills; similar community mobilization and referral, described by one surgeon as the potential to create “total awareness” about maternal and reproductive morbidities; and potential for more effective and complete client management, described by a respondent as the opportunity to provide “total motherhood care [with] no more discrimination.” Examples of potential conflicts include: inadequate and overwhelmed data and QA/QI systems; poor supply chains for POP-specific supplies; difficulty in recruiting and retaining staff owing to remote locations or limited funding; limited infrastructure and space, particularly operating theaters and post-operative wards; and a fear of declining or “crowded out” fistula repairs.

**Table 5:** Potential synergies and conflicts identified by clinicians at fistula centers

Topic	Number of synergies identified	Number of conflicts identified
Human resources	14	6
Access/availability of camps/routine	14	8
Development of surgical skills	16	6
Infrastructure	15	11
Equipment	14	8
Expendable supplies/equipment	12	10
Data management systems	14	8
QA/QI	15	5
Prevention	14	5
Community engagement	13	3
Referral mechanisms	11	4

*QA/QI* quality assurance/improvement

## Discussion

This survey of fistula treatment centers documents an unmet need for POP care and potential for service expansion. Many sites already provide some POP services, but there is scope for growth. This survey revealed a wide variation in access to POP and incontinence care within fistula centers. Many sites that offered POP and incontinence care often did not have the clinical skills required for compartment-based POP, urinary, and rectal incontinence conservative and surgical management. Fistula surgeons reported a high interest in expanding and improving POP services, while simultaneously expressing concerns about facility capacity to expand to include POP services without negative impact on the provision of fistula services. Respondents identified twice as many potential synergies as potential conflicts related to POP and fistula integration.

The study gained strengths through the selection of an online survey methodology. The survey format ensured that a standard set of questions, particularly regarding clinical evaluation skills and specific surgical procedures, was asked of all respondents. The online format also allowed respondents to remain confidential, reducing courtesy bias, and to respond to the questions at their own convenience, essential for surgeons with unpredictable clinical schedules. A limitation of the survey format was the inability to probe responses to more qualitative questions, e.g., related to the synergies and conflicts that may be experienced during integration.

Another important limitation of the study is that it was a cross-sectional survey of respondents from just five countries. The restriction to one respondent per center also limits our ability to ascertain whether the primarily clinical respondents accurately represent the viewpoints of other health facility stakeholders. However, the countries and sites included in this survey constitute a substantial proportion of the global fistula burden and of fistula repairs provided annually. The Global Fistula Map is the largest single attempt to monitor the volume and distribution of fistula services [20]. In 2015, the last year for which data are publicly available, the five countries included in this study accounted for 47% of fistula repairs reported to the Global Fistula Map. The 26 facilities responding to the survey constitute more than a quarter of the 99 facilities reporting fistula repairs in 2015. Therefore, the survey sample represents a significant part of the documented fistula repair landscape, increasing the generalizability of the findings and inferences.

The survey findings affirm prior research and clinician reports indicating that, despite the lack of population-level surveys, the burden of unmet need for POP care remains high in LMICs. The findings suggest that centers offering fistula care can serve as a platform to provide POP and nonfistula incontinence services, with the advantages of such integration outweighing the disadvantages. Such integration is conceptually logical as well, given the impact that both conditions have on the pelvic floor and the overlap in required clinical skills and understanding. With clinical capacity building and support to capitalize on synergies in training, materials, and staffing, these fistula centers could become platforms for integrated, high-quality surgical care. Optimal integration of services requires that clinicians, their teams and their hospitals be staffed and equipped appropriately to engage a minimum acceptable standard of care for integrated services. In centers where fistulas are treated, the clinical and surgical skills required for POP and other types of incontinence already exist, and most would benefit from sustainable integration into academic training centers.

Integration may also enhance the financial and human resource sustainability of fistula care past external or fistula-specific funding—the socioeconomically diverse pool of POP clients may subsidize fistula repair that occurs almost exclusively among women in the lowest socioeconomic strata. Further, service integration enables career longevity and regional skilled professional retention for and among fistula surgeons as the caseload from obstetric causes declines, but remains urgent to address until obstetric fistulas are eradicated.

The potential concerns about integration reported by respondents, however, are substantive and need to be addressed. These concerns suggest that efforts to integrate POP and fistula services, whether at the level of capacity building or service delivery, should include rigorous monitoring to evaluate the effect on fistula services that meet demand among an underserved and impoverished patient population. It is important to note that integration may also enhance fistula services if capacity building efforts are thoughtfully designed with the engagement of the full clinical team at any site.

Pelvic floor trauma associated with obstructed labor can result in a range of consequences including foot-drop and other sacral neuropathies, infertility, vaginal scarring, chronic pain, severe incontinence, genital fistula, and contributing risk for both acute and delayed-onset POP. Thus, integrating POP evaluation and management into established services for fistula care, with integrated training of clinicians and coordinated management of facility resources can be an efficient and important first step toward enabling more appropriate and comprehensive management of the morbidities of obstructed labor beyond obstetric fistulas, deemed the “most disabling of all maternal conditions” [21]. At a time of increased attention to surgical need in low-resource settings, opportunities to reduce disability among women in LMICs through synergistic, sustainable packages of surgical care should be identified and supported. The findings of this survey demonstrate that senior fistula surgeons in diverse LMIC settings believe that the potential for ensuring care for the POP patient can be harnessed within the fistula center setting—thus advancing the global surgery movement’s goal of “access to surgery for 80% of the world’s population by 2030” [4].

## Electronic supplementary material

ESM 1 (PDF 621 kb)
