# Supplementary material for: Demand and capacity to integrate pelvic organ prolapse and genital fistula services in low-resource settings
Source: Int Urogynecol J. 2018 Feb 6;29(10):1509–15. doi: 10.1007/s00192-018-3561-2 (PMC6154024; doi:10.1007/s00192-018-3561-2)
Supplement: Supplementary file 1 — (PDF 621 kb) [file 192_2018_3561_MOESM1_ESM.pdf]

Facility and provider overview

This survey is collecting information from health facilities that conduct surgical fistula repairs with support from the Fistula Care *Plus* project. The survey asks questions about fistula backlog and current demand and capacity for pelvic organ prolapse (POP) services. Data gathered through this survey will assist FC+ in planning program activities in the coming year. We anticipate that it will take 30-45 minutes to complete this survey. The FC+ project sincerely appreciates your contribution to this process.

The survey should be completed by a senior clinician or administrator-in-charge with detailed information about current fistula treatment services. If this person does not have information about current POP needs/services, he or she should consult with clinical colleagues overseeing POP services to complete the survey.

On this first page, please provide background information about this health facility and primary survey respondent.

1. Please provide the name of the primary respondent who is completing this survey on behalf of the facility.

2. Select the primary respondent's cadre/clinician title.

3. What is the primary respondent's relationship to this facility?

4. What is the name of the health facility for which this survey is being completed?

5. What country is this health facility in?

6. Where is your facility located? (Please provide the town/city and state/province)

7. What type of facility is this?

8. Since when has your facility been supported by the EngenderHealth Fistula Care Plus Project and/or Fistula Care Project?

Current fistula services

Please provide information on current fistula services.

9. On average, how many genital fistula patients are documented with a fistula diagnosis at this facility each year?

0-10	11-50	51-100	>100
○	○	○	○

10. How are surgical fistula repair services provided at this facility?

11. How many days per week is an operating theater available for surgical fistula repair services? If the facility does not provide routine surgical fistula repair, please select N/A.

<1 (less than weekly)	1 day	2 days	3 days	4+ days	N/A
○	○	○	○	○	○

12. How many pooled/concentrated efforts for surgical fistula repair are held in a typical year through this facility? If the facility does not conduct pooled/concentrated efforts, please select N/A.

1 per year	2 per year	3 per year	4+ per year	N/A
○	○	○	○	○

13. Provide the names and qualifications (degrees) of any doctors who are ROUTINELY attached to the facility and perform fistula, pelvic organ prolapse (POP) or other urogynecological services. For each doctor, please note which services he/she provides: fistula repair, POP management, and/or other urogynecological services.

14. Provide the names and qualifications (degrees) of mid-level providers (e.g., medical officers) who are ROUTINELY attached to the facility and perform fistula, pelvic organ prolapse (POP) or other urogynecological services. For each mid-level provider, please note which services he/she provides: fistula repair, POP management, and/or other urogynecological services.

15. Provide the names and qualifications (degrees) of any nurses who are ROUTINELY attached to the facility and perform fistula, pelvic organ prolapse (POP) or other urogynecological services. For each nurse, please note which services he/she provides: fistula repair, POP management, and/or other urogynecological services.

Fistula backlog

Please provide information on the backlog of women waiting for fistula surgery.

16. How does this facility keep track of women who are waiting for surgery?

17. How is the information (e.g., list or database) on women waiting for surgery updated? Please include frequency of update in your response.

18. Is there a separate list/tracking for women who are waiting "in situ" (e.g., at a waiting home)

Yes, separate list for "in situ"	No separate list for "in situ"	No list at all of women waiting
○	○	○

19. Is there a separate list/tracking for women who are waiting and have returned to their communities?

Yes, separate list for women who have returned to communities	No separate list for women who have returned to communities	No list at all of women waiting
○	○	○

20. What **percentage** of women waiting for fistula repair are "in situ" vs. returned back home? Please include a % in your answer. If this value is not known, please write "Don't Know."

21. What is the mechanism used to determine the number of women waiting who are currently eligible for fistula surgery, vs. those who are waiting/there for other reasons? *Other reasons include: women deemed incurable/women with persistence incontinence, "social holds," staged repairs, other obstructed labor sequelae (e.g., impaired ambulation, vaginal obliteration, hematometra, colostomy, severe mental health).*

22. Of the women waiting, **what percentage** are currently eligible for fistula surgery? Please write a % in your answer. If this value is not known, please write "Don't Know." Please see question 21 for conditions that would make a woman not eligible for surgery.)

23. How many women are currently waiting for fistula surgery at this facility? If no women are waiting for fistula surgery, please select N/A.

0-10	11-50	51-100	>100	N/A
○	○	○	○	○

24. How are women who are waiting for fistula repair contacted/scheduled to come back for treatment?

25. From year to year, is your facility's total backlog of women waiting for fistula surgery typically increasing, decreasing, or remaining the same?

26. Please describe the reasons for the trend in your facility's backlog provided in Question 25.

POP service demand

Please provide information about the demand for POP services at this facility.

27. On average, how many POP patients arrive to your gynecological services/program each month? Please select N/A if there are typically no POP patients

0-10	11-50	51-100	>100	N/A
○	○	○	○	○

28. How many POP patients arrive at an average fistula screening effort (e.g., diagnostic camp, community outreach activites, facility-based screening campaign). Please select N/A if there are typically no POP patients.

0-10	11-50	51-100	>100	N/A
○	○	○	○	○

29. What do you estimate to be the ratio of patients seeking fistula care to those seeking POP care? (For example, if there are half as many patients arriving with fistula as patients arriving with POP, the ratio is 1:2)

30. Is there a need for surgical and/or non-surgical POP treatment at this health facility?

* 31. Is your health facility currently providing treatment for patients who arrive seeking POP services?

32. Is the need for POP treatment at this facility fully met?

Please provide any comments on whether/how POP treatment need is met at this facility.

33. Do you refer patients who arrive seeking POP services to another facility for treatment?

Yes, refer all POP patients	Yes, refer some POP patients	No, refer no POP patients
○	○	○

34. If you refer POP patients to another facility/facilities for treatment, please describe for which services you refer out, where you send them, and the distance of this facility/facilities from your site. If you do not refer for POP treatment, **please write N/A.**

POP evaluation

Please provide information about how this facility's clinical team assesses POP cases.

35. Is your team performing compartment-based assessment of POP?

36. Is your team grading POP

37. Describe routine procedures related to history-taking and evaluation of lower urinary tract symptoms (LUTS) in POP patients.

	Routinely - all/near all patients	Sometimes	No
Does your team collect history on LUTS in POP patients?	○	○	○
Does your team use bladder diary in evaluation of LUTS?	○	○	○
Does your team use pad test in evaluation of incontinence?	○	○	○

38. Does your team have the skills and materials for the following bladder function testing procedures?

	Skills	Materials
Simple cystometrics testing		
Multichannel urodynamics testing		

39. How does your team apply simple cystometrics testing of LUTS in POP patients? If you do not have the skills or materials for simple cystometrics, please select N/A.

	Regularly performed	Sometimes performed	Not performed	N/A
Testing of LUTS	○	○	○	○
Testing of urinary incontinence	○	○	○	○
Testing of voiding dysfunction	○	○	○	○

40. How does your team apply multichannel urodynamics testing of LUTS in POP patients? If you do not have the skills or materials for multichannel urodynamics, please select N/A.

	Regularly performed	Sometimes performed	Not performed	N/A
Testing of LUTS	○	○	○	○
Testing of urinary incontinence	○	○	○	○
Testing of voiding dysfunction	○	○	○	○

41. How does your team screen for occult stress urinary incontinence in POP patients?

[▾]

Non-surgical POP management

Please provide information on the services you provide for non-surgical management of POP.

42. Is your team providing pelvic floor physical therapy (i.e., Kegel exercises) instructions to POP patients?

Average monthly number of POP patients receiving physical therapy

43. What proportion of POP patients receiving pelvic floor physical therapy instructions return for follow-up evaluation of POP? If you do not provide physical therapy instruction, please select N/A.

All	Most	Some	None	N/A
○	○	○	○	○

44. Is your team providing pessary management for POP patients?

Average monthly number of POP patients receiving pessaries

45. Do you have reliable pessary procurement? If you do not provide pessary management of POP, please select N/A.

Yes, very reliable procurement	Occasional stockouts	Frequent stockouts	No, almost never have pessaries	N/A
○	○	○	○	○

46. What type of pessary do you use? If you do not provide pessary management of POP, please select N/A.

Simple ring	Plate ring	Other (please specify below)	N/A
○	○	○	○

Other type of pessary

Surgical POP managent - Cystoscopy

Please provide information on the services your team provides using cystoscopy.

47. Does your team have access to cystoscopy?

48. How often does your team provide the following procedures using cystoscopy? If you do not have cystoscopy or do not provide a service, please select N/A.

	0-5 per month	6-20 per month	21+ per month	N/A
Cystoscopy diagnostic	○	○	○	○
Cystoscopy ureteric catheterization	○	○	○	○
Cystoscopy – other (please describe below)	○	○	○	○

Please describe other procedures provided using cystoscopy.

Surgical POP management - vaginal surgery

Please provide information on the type and number of vaginal surgery POP procedures your team provides.

49. How often does your team provide the following **vaginal** apex compartment repairs? Please select N/A if the facility does not conduct a specific procedure.

	0-5 per month	6-20 per month	21+ per month	N/A
Uterosacral cuff or vault suspension	○	○	○	○
Uterosacral hysteropexy	○	○	○	○
Sacrospinous vault or uterine suspension	○	○	○	○
Enterocele repair	○	○	○	○
Other (please describe below)	○	○	○	○

Please describe any other apex compartment procedures provided.

50. How often does your team provide the following **vaginal** anterior compartment repairs? Please select N/A if the facility does not conduct a specific procedure.

	0-5 per month	6-20 per month	21+ per month	N/A
Anterior colporrhaphy cystocele repair	○	○	○	○
Vaginal paravaginal cystocele repair	○	○	○	○
Other (please describe below)	○	○	○	○

Please describe any other anterior compartment procedures provided.

51. How often does your team provide the following **vaginal** posterior compartment repairs? Please select N/A if the facility does not conduct a specific procedure.

	0-5 per month	6-20 per month	21+ per month	N/A
Levatorplasty rectocele repair	○	○	○	○
Site-specific rectocele repair	○	○	○	○
Perineorrhaphy	○	○	○	○
Perineoplasty	○	○	○	○
Other (please describe below)	○	○	○	○

Please describe any other posterior compartment procedures provided.

Surgical POP management - abdominal surgery

Provide information on the number and type of other abdominal surgery POP procedures your team provides.

52. How often does your team provide the following abdominal apex compartment repairs? Please select N/A if the facility does not conduct a specific procedure.

	0-5 per month	6-20 per month	21+ per month	N/A
Uterosacral vault suspension	○	○	○	○
Uterosacral hysteropexy	○	○	○	○
Sacro-colpopexy	○	○	○	○
Sacro-hysteropexy	○	○	○	○
Other (please describe below)	○	○	○	○

Please describe any other apex compartment procedures provided.

53. How often does your team provide the following abdominal anterior compartment repairs? Please select N/A if the facility does not conduct a specific procedure.

	0-5 per month	6-20 per month	21+ per month	Not provided/not applicable
Paravaginal repair cystocele	○	○	○	○
Other (please describe below)	○	○	○	○

Please describe any other anterior compartment procedures provided.

Surgical POP management - surgery for concomitant urinary incontinence

Please describe whether your team provides the following procedures for POP patients with concomitant urinary incontinence.

54. How often does your team provide the following vaginal procedures? Please select N/A if the facility does not conduct a specific procedure.

	0-5 per month	6-20 per month	21+ per month	N/A
Urethropexy (Kelly Plication)	○	○	○	○
Other (please describe below)	○	○	○	○

Please describe any other vaginal procedures provided.

55. How often does your team provide the following abdominal procedures? Please select N/A if the facility does not conduct a specific procedure.

	0-5 per month	6-20 per month	21+ per month	Not provided/not applicable
Urethropexy (Burch Procedure)	○	○	○	○
Other (please describe below)	○	○	○	○

Please describe any other abdominal procedures provided.

56. How often does your team provide the following combined abdominal-vaginal procedures? Please select N/A if the facility does not conduct a specific procedure.

	0-5 per month	6-20 per month	21+ per month	Not provided/not applicable
Rectus fascia autologous sling	◯	◯	◯	◯
Fascia lata autologous sling	◯	◯	◯	◯
Other (please describe below)	◯	◯	◯	◯

Please describe any other combined abdominal-vaginal procedures provided.

Surgical POP management - surgery for concomitant rectal incontinence

Please describe whether your team provides the following procedures for POP patients with concomitant rectal incontinence.

57. How often does your team provide the following vaginal procedures? Please select N/A if the facility does not conduct a specific procedure.

	0-5 per month	6-20 per month	21+ per month	N/A
Anal sphincteroplasty	○	○	○	○
Other (please describe below)	○	○	○	○

Please describe any other vaginal procedures provided.

[ ]

Capacity to expand POP evaluation and management

Please provide information on this health facility's ability to expand POP services.

58. Training for **surgical** POP evaluation and management (E&M)

	No capacity/interest	Some capacity/interest	A lot of capacity/interest
Is there team **capacity** to engage fistula surgeons and nurses in training for surgical POP E&M?	○	○	○
Is there team **interest** in engaging fistula surgeons and nurses in training for surgical POP E&M?	○	○	○
Is there institutional **capacity** to engage fistula surgeons and nurses in training for surgical POP E&M?	○	○	○
Is there institutional **interest** in engaging fistula surgeons and nurses in training for surgical POP E&M?	○	○	○

Please provide any additional comments on team/institutional capacity and interest in training for surgical POP E&M. If you already provide surgical POP E&M, please discuss how you would like to expand these services or improve their quality.

59. Training for **non-surgical** POP E&M

	No capacity/interest	Some capacity/interest	Much capacity/interest
Is there team **capacity** to engage fistula surgeons and nurses in training for non-surgical POP E&M?	○	○	○
Is there team **interest** in engaging fistula surgeons and nurses in training for non-surgical POP E&M?	○	○	○
Is there institutional **capacity** to engage fistula surgeons and nurses in training for non-surgical POP E&M?	○	○	○
Is there institutional **interest** in engaging fistula surgeons and nurses in training for non-surgical POP E&M?	○	○	○

Please provide any additional comments on team/institutional capacity and interest in training for non-surgical POP E&M. If you already provide non-surgical POP services, please discuss how you would like to expand these services or improve their quality.

60. Does your team have access to referral for physical therapy?

61. Does the physical therapy group have female therapists interested in learning pelvic floor physical therapy? If there is on physical therapy group, please select N/A.

Yes - interested female therapists	No - female therapists not interested	No - no female therapists	N/A
○	○	○	○

POP integration synergies and conflicts

When integrating fistula and POP services, there may be synergies (advantages, opportunities, strengths) and conflicts (barriers, weaknesses, threats). For each of the topics below, please name up to three or synergies and up to three conflicts. If there are no conflicts or synergies, please write NONE.

62. Numbers/deployment of service providers?

Synergies

Conflicts

63. Access and availability of routine and/or concentrated effort services for clients?

Synergies

Conflicts

64. Development of surgical skills?

Synergies

Conflicts

65. Infrastructure?

Synergies

Conflicts

66. Equipment?

Synergies

Conflicts

67. Expendable supplies and medications?

Synergies

Conflicts

68. Data management systems?

Synergies

Conflicts

69. Quality assurance processes/quality improvement interventions?

Synergies

Conflicts

70. Prevention interventions?

Synergies

Conflicts

71. Community engagement?

Synergies

Conflicts

72. Referral mechanisms?

Synergies

Conflicts
